# Multimodal ultrasonography in the diagnosis and treatment of chronic thyrotoxic myopathy: a prospective study

**DOI:** 10.3389/fendo.2025.1590294

**Published:** 2025-09-11

**Authors:** Roumei Wang, Rui Huang, Shien Fu, Yunxia Tang, Yaoli Liu

**Affiliations:** ^1^ Department of Medical Ultrasound, The First Affiliated Hospital of Guangxi Medical University, Nanning, Guangxi, China; ^2^ Department of Spine Osteopathia, The First Affiliated Hospital of Guangxi Medical University, Guangxi Medical University, Nanning, Guangxi, China; ^3^ College of Public Hygiene of Guangxi Medical University, Nanning, Guangxi, China; ^4^ Department of Endocrinology, The First Affiliated Hospital of Guangxi Medical University, Nanning, Guangxi, China; ^5^ Department of Ultrasound, Maternal and Child Health Hospital of Guangxi Zhuang Autonomous Region, Nanning, Guangxi, China

**Keywords:** chronic thyrotoxic myopathy, shear wave elastography, rectus femoris, thyroid gland, ultrasonography

## Abstract

**Objective:**

This study integrates high-frequency ultrasonography with shear wave elastography (SWE) to perform a multimodal quantitative assessment of the rectus femoris and thyroid tissue architecture in patients with chronic thyrotoxic myopathy (CTM). A novel predictive model for CTM progression was developed by quantitatively assessing biomechanical parameters, including muscle and thyroid parenchymal stiffness, providing an objective imaging-based foundation for early disease intervention.

**Methods:**

This prospective study enrolled 75 patients with hyperthyroidism and 53 healthy controls. The biomechanical parameters of the rectus femoris and thyroid tissue were quantitatively assessed using SWE. Receiver operating characteristic (ROC) curve analysis was performed to evaluate the predictive efficacy of rectus femoris and thyroid tissue elasticity parameters for CTM diagnosis. A multivariate logistic regression model was developed to investigate the association between muscle stiffness, thyroid stiffness, and CTM risk. Additionally, patients with CTM received pharmacotherapeutic interventions, and clinical indexes were systematically monitored along with dynamic changes in thyroid and rectus femoris correlation parameters before and after treatment to evaluate therapeutic efficacy.

**Results:**

The CTM group exhibited a significantly lower mean Young’s modulus (E-mean) of the rectus femoris than the non-CTM and healthy groups (*p* < 0.01). In contrast, the CTM group had a significantly higher thyroid E-mean than the other groups (*p* < 0.01). Moreover, the rectus femoris E-mean was positively correlated with grip strength (r = 0.437, *p* < 0.01) and lower limb fatigue test (r = 0.247, *p* = 0.042) in patients with CTM. ROC curve analysis revealed that the combined diagnostic approach achieved the highest sensitivity and specificity for CTM, with an area under the curve of 92.5%. A multivariate logistic regression model revealed that, after adjusting for confounding factors, rectus femoris E-mean and thyroid E-mean were significant predictors of CTM. Following treatment with antihyperthyroid drugs, significant improvements were observed in thyroid hormone levels, upper and lower limb muscle function tests, and muscle-related parameters compared with their pretreatment levels, with all differences reaching statistical significance (*p* < 0.01).

**Conclusion:**

By quantitatively assessing the morphological and biomechanical characteristics of the rectus femoris and thyroid gland, multimodal ultrasonography can provide a reliable imaging basis for the early prediction and therapeutic monitoring of CTM.

## 1 Introduction

Chronic thyrotoxic myopathy (CTM), a neuromuscular disorder, is characterized by progressive weakness of the proximal muscle groups (1, 2). Although CTM exhibits typical clinical features, certain patients may not manifest the common symptoms of hyperthyroidism, which complicates early diagnosis and may even result in misdiagnosis as a primary myopathy (3). If CTM is not promptly recognized and treated, it may progress to irreversible muscle damage and functional impairment, significantly affecting the quality of life of patients. Therefore, establishing an efficient early diagnostic strategy and implementing long-term follow-up management are clinically significant for mitigating the risk of complications and enhancing patient prognosis. To achieve this, developing efficient noninvasive assessment tools for comprehensively evaluating skeletal muscle status is crucial (4). Ultrasonography plays an increasingly important role in the diagnosis, differential diagnosis, and treatment guidance of musculoskeletal disorders. Its noninvasive nature, real-time capability, and high resolution make it a potentially valuable tool for evaluating skeletal muscle structure and function in patients with CTM.

The rapid development of ultrasonic elastography (UE) has also offered new insights into musculoskeletal disease assessment. UE can sensitively detect early microstructural changes in muscle tissues by quantifying tissue stiffness, demonstrating considerable advantages in early diagnosis, disease staging, and treatment monitoring of the tendons, muscles, nerves, and ligaments (5). UE-based shear wave elastography (SWE) provides real-time quantitative measurements of Young’s modulus of the muscle tissue, offering an objective reflection of changes in tissue stiffness, thereby providing crucial evidence for disease diagnosis and treatment (6–8).

The use of SWE has been increasingly explored in thyroid research. SWE can quantitatively assess changes in thyroid tissue stiffness, offering objective evidence for the diagnosis, treatment, and follow-up of diffuse thyroid diseases (9–11). However, research on the application of SWE in patients with CTM is limited, particularly regarding the simultaneous evaluation of rectus femoris and thyroid stiffness, which has not yet been reported.

Therefore, this study is the first to apply SWE for the simultaneous quantitative assessment of the rectus femoris and thyroid tissues in patients with CTM. This study aimed to comprehensively analyze changes in muscle and thyroid stiffness using multimodal ultrasonographic techniques, address the limitations of conventional diagnostic methods, and provide new imaging tools to support the early prediction, diagnosis, and therapeutic monitoring of CTM.

## 2 Materials and methods

### 2.1 Study design and inclusion criteria

This study prospectively screened 350 potential participants and ultimately enrolled 128 individuals (males, 49; females, 79) who met the study criteria. The cohort comprised 75 patients with hyperthyroidism (hyperthyroidism group) and 53 healthy controls (control group). The hyperthyroidism group was further divided into a CTM subgroup (n = 35) and a non-CTM subgroup (n = 40). The primary reason for exclusion was the inability to complete follow-up.

### 2.2 Diagnostic criteria for CTM

The diagnosis of CTM was based on the following criteria:

1. Hyperthyroidism confirmed by laboratory tests and clinical manifestations.
2. Symmetric proximal muscle weakness, with or without muscle atrophy.
3. Electromyography revealing myopathic features or muscle biopsy confirming myopathic changes.
4. Exclusion of neuromuscular disorders attributable to other causes.
5. Significant improvement in muscle weakness symptoms following antihyperthyroid treatment.

### 2.3 Medical history collection and skeletal muscle function assessment

A detailed medical history was collected, documenting the demographic and clinical characteristics of the participants, including age, sex, height, weight, blood pressure, and heart rate. All patients with CTM underwent thyroid function tests and routine biochemical assays before and after treatment.

Skeletal muscle strength and function analysis encompassed grip strength measurement, fixed-distance walking test, upper limb endurance test, lower limb endurance test, and squat–stand test. Grip strength was assessed using an electronic dynamometer, with participants comfortably seated or standing and gripping the handle with their right hand at maximal force; the result was recorded once the value stabilized, and the procedure was repeated for the left hand. Before each measurement, the dynamometer was calibrated to zero, and each hand was alternately tested three times, with the highest value (in kg) recorded as the grip strength. For the fixed-distance walking test, participants were instructed to walk 9 m at their usual pace without slowing down, and the time taken was recorded; two measurements were performed, with the fastest time recorded to an accuracy of 0.01 m/s. For the upper limb endurance test, participants stood with their arms raised to shoulder height, and the test was terminated when the arms dropped below 90° relative to the torso or when the position was maintained for >120 s, with the time recorded. For the lower limb endurance test, participants lay supine on an examination bed with both legs raised to 90°, performing hip and knee flexion. The test was terminated when the legs bent or dropped below 90° or when the position was maintained for >120 s, with the time recorded. For the squat–stand test, participants stood with their arms extended forward at shoulder height, squatted until the hips were below the knees, and subsequently stood up. The test was terminated after 10 repetitions or when the total time was 30 s. When participants were unable to stand up with their arms extended forward, they were allowed to use their hands to support their knees, and the test was terminated when they could not stand up within 5 s, with the time recorded.

### 2.4 Ultrasonographic measurement methodology

Ultrasonography was performed using the Mindray Resona7 or Wisonic Clover50 color Doppler ultrasonography diagnostic systems equipped with high-frequency linear array probes (4–15 MHz). Both musculoskeletal and thyroid modes were used to scan and measure the rectus femoris muscle and thyroid gland parameters. The SWE technique was employed, with the regions of interest set on long-axis images. The probe was held steady until the color fill within the sampling box stabilized and the image reliability exceeded 90%, after which the image was frozen and saved. Subsequently, the quantitative analysis system was activated, and the circle diameter of the elasticity parameter was adjusted to 5 mm. The mean Young’s modulus (E-mean) and elasticity standard deviation (Esd) were used as indicators for analyzing muscle and thyroid stiffness. The same sonographer performed all ultrasonography procedures, and at least three images were saved for each standard plane. Each ultrasonographic parameter was measured three times, and the average value was calculated to ensure data accuracy and reliability. The rectus femoris was selected as the target muscle in this study because its superficial location, well-defined orientation, and clear boundaries facilitate ultrasound localization and repeated measurements.

### 2.5 Statistical analysis

All data were statistically analyzed using Statistical Package for the Social Sciences (version 26, IBM, Armonk, NY, USA). Categorical data were expressed as frequencies, and intergroup comparisons were performed using the chi-square test. The normality and homogeneity of variance were tested for continuous data. Data conforming to normal distribution were expressed as means ± standard deviations, and comparisons among multiple groups were conducted using one-way analysis of variance, with the LSD *t*-test for homogeneous variance and Tamhane’s T2 test for heterogeneous variance. Data not conforming to normal distribution were presented as medians (interquartile ranges), and intergroup comparisons were performed using the Mann–Whitney *U* test or Kruskal–Wallis H test. Receiver operating characteristic (ROC) curves were plotted for parameters exhibiting significant differences to analyze the optimal threshold, sensitivity, specificity, and area under the curve (AUC) of ultrasonographic parameters for predicting CTM. To evaluate the association between clinical indicators and ultrasonographic measurements in patients with CTM, correlation analysis was performed using Pearson’s correlation coefficient (for normally distributed data) or Spearman’s rank correlation (for non-normally distributed data). Binary logistic regression analysis was used to identify the factors associated with CTM. A *p*-value < 0.05 was considered statistically significant.

## 3 Results

### 3.1 Comparison of clinical data before treatment

This study enrolled 128 participants who were divided into the CTM (n = 35), non-CTM (n = 40), and healthy control (n = 53) groups. No significant differences were observed in baseline characteristics, including age, sex, and height, between the groups (*p* > 0.05). However, significant differences were observed in metabolic and hormone-related indicators, particularly pulse pressure, resting heart rate, and thyroid parameters. The CTM and non-CTM groups showed significantly higher thyroid hormone (free triiodothyronine [FT3], free thyroxine [FT4] and related antibody (thyroglobulin antibody, thyrotropin receptor antibody [TRAB], and thyroid peroxidase antibody) levels than the healthy control group (*p* < 0.001, **Table 1**).

**Table 1:** Comparison of baseline characteristics of participants.

Parameter	Healthy group (n = 53)	Non-CTM group (n = 40)	CTM group (n = 35)	*p*
Age (years)	32.00 (25.00, 41.25)	33.00 (27.00, 45.50)	31.00 (24.25, 39.75)	0.496
Sex				0.595
Male	21 (39.6%)	17 (42.5%)	11 (31.4%)	
Female	32 (60.4%)	23 (57.5%)	24 (68.6%)	
Height (m)	1.60 (1.55, 1.68)	1.60 (1.57, 1.66)	1.60 (1.57, 1.64)	0.887
Weight (kg)	58.00 ± 9.72	56.39 ± 9.98	53.07 ± 8.52	0.087
BMI (kg/m^2^)	21.65 ± 4.12	21.70 ± 3.25	20.53 ± 2.62	0.083
Pulse pressure (mmHg)	42.00 (35.00, 47.50)	48.00 (43.00, 59.25)	54.00 (47.25, 61.50)	<0.001^*^
Resting heart rate (bpm)	70.00 (62.75, 76.00)	95.00 (80.25, 104.75)	107.00(100.25, 120.75)	<0.001^*^
Waist-to-hip ratio	0.86 ± 0.07	0.86 ± 0.06	0.85 ± 0.05	0.580
FT3 (pmol/L)	4.95 (4.22, 5.34)	15.59 (11.19, 28.09)	30.78 (23.46, 45.89)	<0.001^*^
FT4 (pmol/L)	11.19 (10.18, 11.78)	37.99 (28.38, 51.88)	63.65 (48.28, 76.73)	<0.001^*^
TSH (mIU/L)	2.16 (1.01, 3.44)	0.01 (0.01, 0.01)	0.01 (0.01, 0.01)	<0.001^*^
TGAB (IU/mL)	5.00 (3.99, 10.50)	24.00 (10.04, 35.44)	34.74 (14.17, 62.87)	<0.001^*^
TRAB (IU/L)	0.25 (0.25, 0.25)	10.75 (3.05, 18.68)	15.55 (9.99, 24.13)	<0.001^*^
TPOAB (IU/mL)	3.12 (1.02, 7.24)	633.50 (165.25, 917.25)	723.00 (329.00, 851.25)	<0.001^*^

^*^Represents *p* < 0.05; CTM, chronic thyrotoxic myopathy; BMI, body mass index; FT3, free triiodothyronine; FT4, free thyroxine; TSH, thyroid-stimulating hormone; TGAB, thyroglobulin antibody; TRAB, thyrotropin receptor antibody; TPOAB, thyroid peroxidase antibodies.

### 3.2 Quantification of rectus femoris and thyroid stiffness using the SWE technology

**Figure 1:**
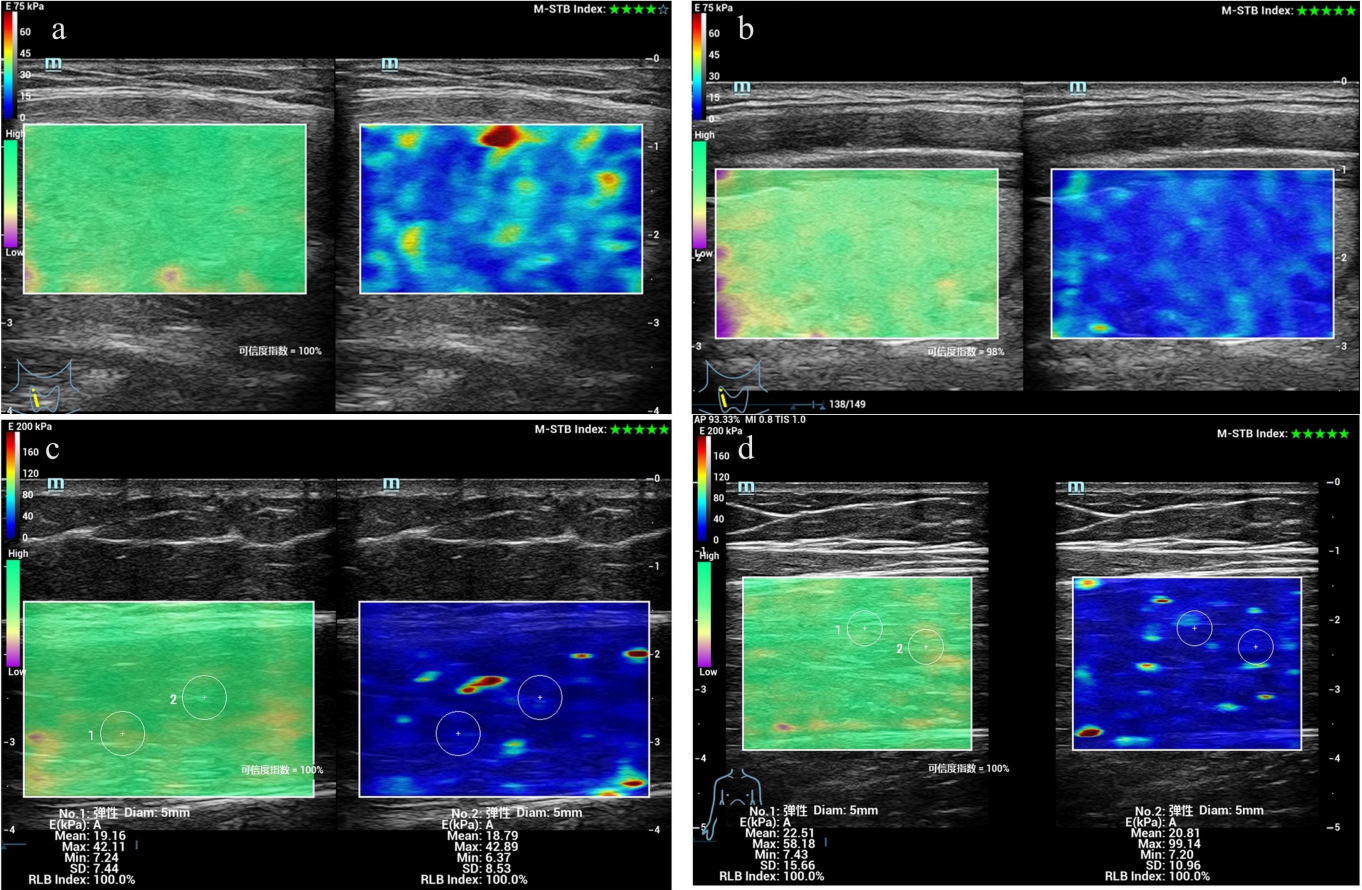
Measurement of Young’s modulus values in the rectus femoris and thyroid gland of patients with CTM using SWE. **(a)** Thyroid elastogram of a 25-year-old female patient with CTM. **(b)** Thyroid elastogram of a 26-year-old healthy male participant. **(c)** Rectus femoris elastogram of a 36-year-old female patient without CTM. **(d)** Thyroid elastogram of a 29-year-old healthy male participant. Abbreviations: CTM, chronic thyrotoxic myopathy; SWE, shear wave elastography.

We compared the differences in several parameters of the thyroid gland and rectus femoris muscle among the CTM, non-CTM, and healthy control groups. The CTM group exhibited significantly higher peak systolic velocity (PS) and end diastolic velocity (ED) of the superior thyroid artery and a higher E-mean of the thyroid gland than the non-CTM and healthy control groups. Patients with hyperthyroidism exhibited thyroid enlargement due to hyperplasia, hypertrophy, hyperemia, and hyperfunction of the thyroid cells, with the CTM and non-CTM groups showing significantly larger thyroid volumes than the healthy control group (*p* < 0.001). In contrast, the E-mean and Esd of the rectus femoris muscle were significantly lower in the CTM group than in the other two groups (*p* < 0.001). Furthermore, the superior thyroid artery pulsatility index (PI) and resistance index (RI) showed no significant differences across the groups (**Figure 1**, **Table 2**).

**Table 2:** Comparison of ultrasonography elastography parameters and thyroid ultrasonography indicators among participants.

Parameter	Healthy group (n = 53)	Non-CTM group (n = 40)	CTM group (n = 35)	*p*
Thyroid E-mean (kPa)	17.27 (16.29, 18.03)	18.21 (17.18, 19.89)	18.80 (18.37, 20.65)	<0.001^*^
Thyroid Esd	3.83 (2.98, 4.23)	3.06 (2.77, 3.74)	2.87 (2.70, 3.34)	<0.001^*^
Rectus Femoris E-mean (kPa)	22.70 ± 2.880	19.65 ± 2.474	15.67 ± 2.271	<0.001^*^
Rectus femoris Esd	9.03 (8.13, 10.22)	7.53 (6.84, 9.35)	6.22 (5.41, 6.90)	<0.001^*^
PS (cm/s)	16.10 (11.38, 20.07)	43.75 (30.61, 58.36)	63.40 (57.95, 76.70)	<0.001^*^
ED (cm/s)	6.68 (4.77, 8.74)	19.80 (12.11, 24.81)	28.65 (25.07, 33.62)	<0.001^*^
PI	0.85 (0.79, 0.96)	0.93 (0.82, 1.06)	0.83 (0.75, 0.95)	0.065
RI	0.56 (0.53, 0.59)	0.57 (0.55, 0.61)	0.55 (0.53, 0.61)	0.424
Thyroid volume (mL)	10.53 (8.04, 13.74)	19.39 (15.68, 28.22)	27.20 (22.76, 32.44)	<0.001^*^

^*^Represents *p* < 0.05; CTM, chronic thyrotoxic myopathy; PS, peak systolic velocity; ED, end diastolic velocity; PI, pulsatility index; RI, resistance index; E-mean, mean of Young’s modulus; Esd, elastic data dispersion.

### 3.3 Diagnostic value of ultrasonographic elastography indicators for CTM

**Figure 2:**
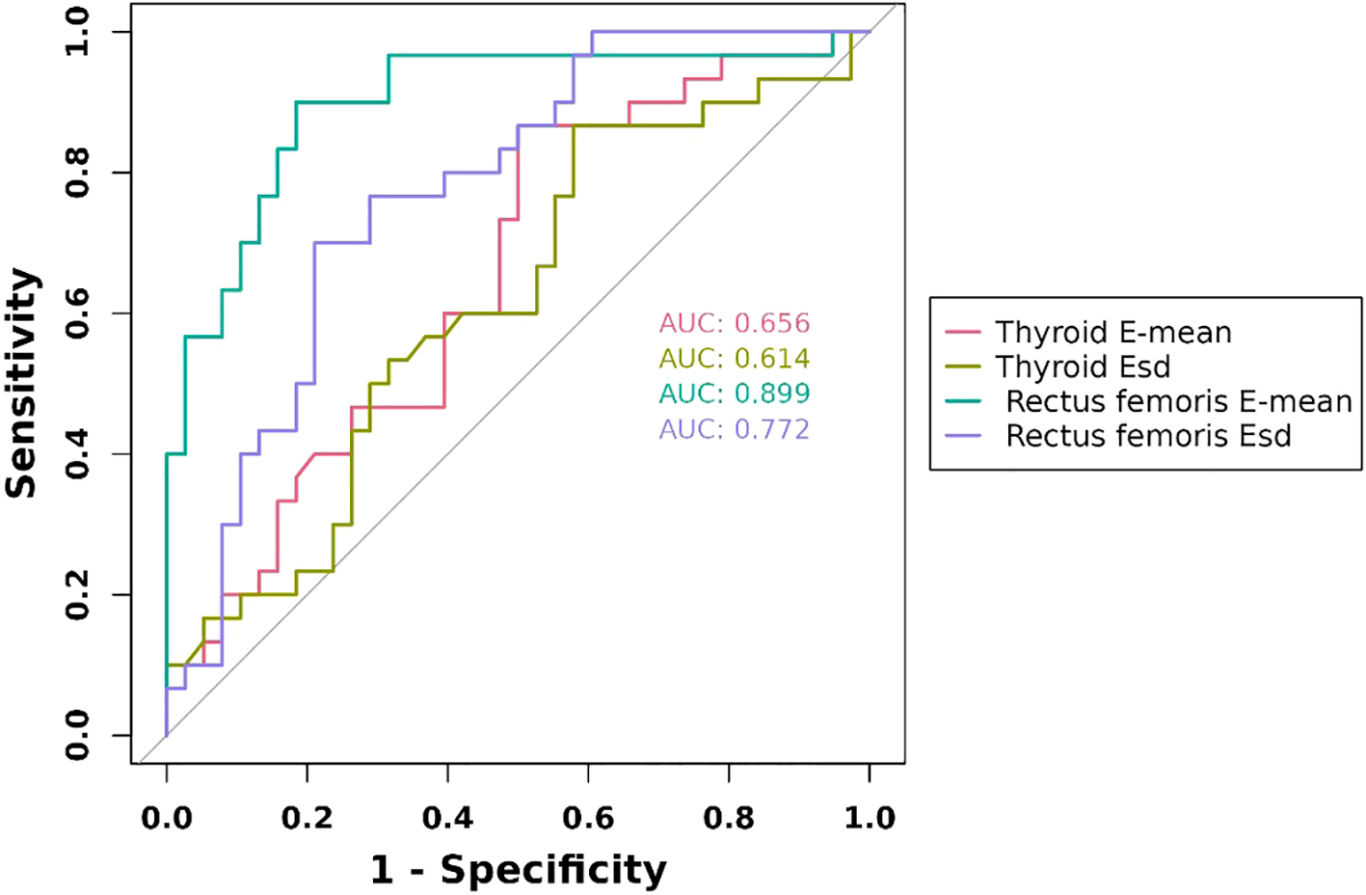
ROC curves of ultrasonography elastography indicators. E-mean, mean of Young’s modulus; Esd, Elastic data dispersion; ROC, receiver operating characteristic.

**Figure 3:**
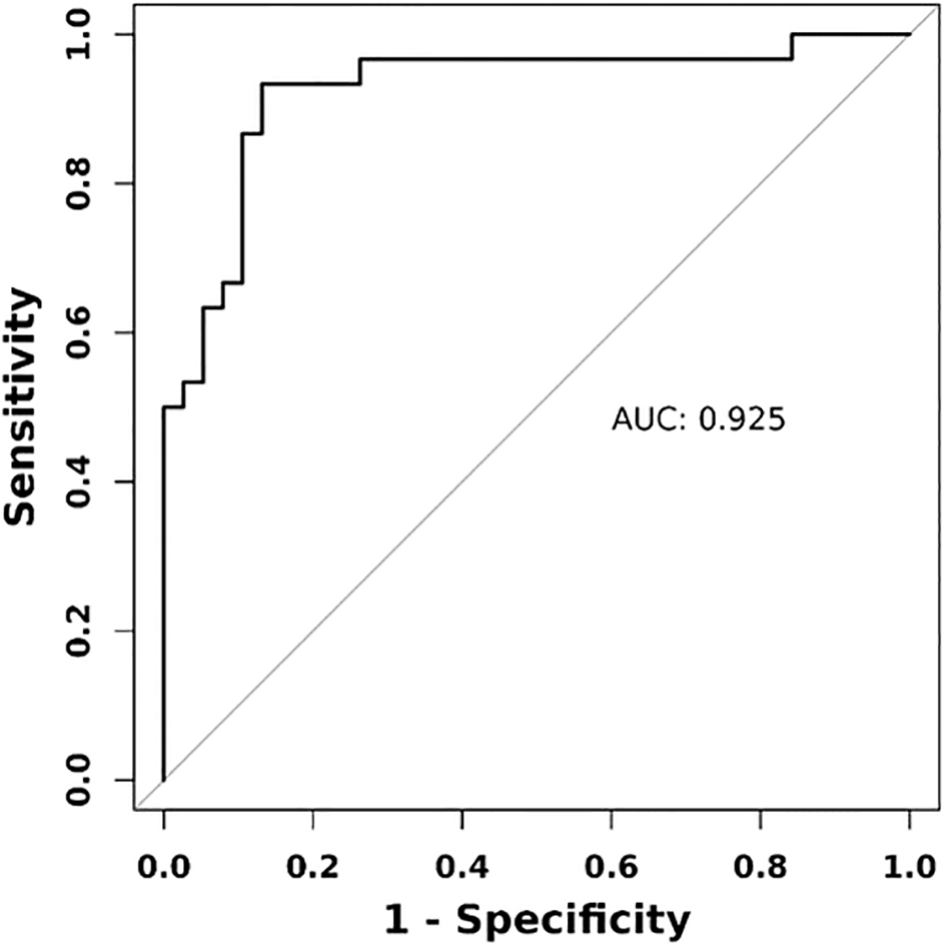
ROC curve of combined diagnostic approach using ultrasonography elastography indicators. Combined indicators encompass rectus femoris E-mean, rectus femoris Esd, thyroid E-mean, and thyroid Esd.

We evaluated the predictive performance of several indicators. The rectus femoris E-mean had the strongest predictive ability, with a 0.899 AUC, 90.0% sensitivity, and 81.6% specificity. The rectus femoris Esd had an AUC of 0.772, with higher specificity (78.9%) and lower sensitivity (70.0%). Thyroid-related indicators exhibited weaker predictive performance, with the thyroid E-mean showing an AUC of 0.656, with higher sensitivity (86.7%) and lower specificity (50.0%). The combined diagnostic approach significantly enhanced predictive performance, achieving an AUC of 92.5%, outperforming individual indicators. These findings suggest that the combined use of the rectus femoris E-mean and thyroid E-mean can significantly enhance predictive accuracy, offering more reliable clinical evidence (**Figures 2**, **3**).

### 3.4 Analysis of factors influencing CTM development

We evaluated the effects of various factors using four regression models. The results revealed that the thyroid E-mean was consistently identified as a risk factor for CTM across all models (odds ratio [OR] = 1.55–1.80, *p* < 0.05), whereas the rectus femoris E-mean was consistently identified as a protective factor (OR = 0.41–0.47, *p* < 0.05). In Model 4, Esd also emerged as a protective factor (OR = 0.13, *p* = 0.045). The thyroid E-mean and rectus femoris E-mean were statistically significant across all models, underscoring their core predictive value (**Table 3**).

**Table 3:** Analysis of factors influencing CTM development.

Model	Parameter	B	OR	95% CI	p-value
Model 1	Thyroid E-mean	0.436	1.55	1.05, 2.27	0.026^*^
	Thyroid Esd	−0.706	0.49	0.13, 1.91	0.307
	Rectus femoris E-mean	−0.763	0.47	0.31, 0.71	<0.001^*^
	Rectus femoris Esd	−0.157	0.85	0.50, 1.47	0.568
Model 2	Thyroid E-mean	0.453	1.57	1.08, 2.29	0.018^*^
	Thyroid Esd	−0.949	0.39	0.10, 1.58	0.185
	Rectus femoris E-mean	−0.867	0.42	0.26, 0.68	<0.001^*^
	Rectus femoris Esd	−0.060	0.94	0.55, 1.62	0.830
Model 3	Thyroid E-mean	0.490	1.63	1.08, 2.47	0.020^*^
	Thyroid Esd	−1.433	0.24	0.05, 1.15	0.074
	Rectus femoris E-mean	−0.883	0.41	0.23, 0.74	0.003^*^
	Rectus femoris Esd	−0.073	0.93	0.50, 1.71	0.815
Model 4	Thyroid E-mean	0.585	1.80	1.06, 3.04	0.029^*^
	Thyroid Esd	−2.078	0.13	0.02, 0.96	0.045^*^
	Rectus femoris E-mean	−0.892	0.41	0.21, 0.81	0.010^*^
	Rectus femoris Esd	−0.269	0.76	0.35, 1.66	0.496

^*^Represents p < 0.05; Model 1 is adjusted for thyroid E-mean, thyroid Esd, rectus femoris E-mean, and rectus femoris Esd. Model 2 is adjusted for age, sex, BMI, thyroid E-mean, thyroid Esd, rectus femoris E-mean, and rectus femoris Esd. Model 3 is adjusted for age, sex, BMI, PS, ED, thyroid E-mean, thyroid Esd, rectus femoris E-mean, and rectus femoris Esd. Model 4 is adjusted for age, sex, BMI, PS, ED, thyroid E-mean, thyroid Esd, rectus femoris E-mean, rectus femoris Esd, average walking speed (m/s), and squat–stand test time. Abbreviations: CTM, chronic thyrotoxic myopathy; E-mean, mean of Young’s modulus; Esd, elastic data dispersion.

### 3.5 Correlations between skeletal muscle function, strength tests, and elasticity indicators in patients with CTM

**Figure 4:**
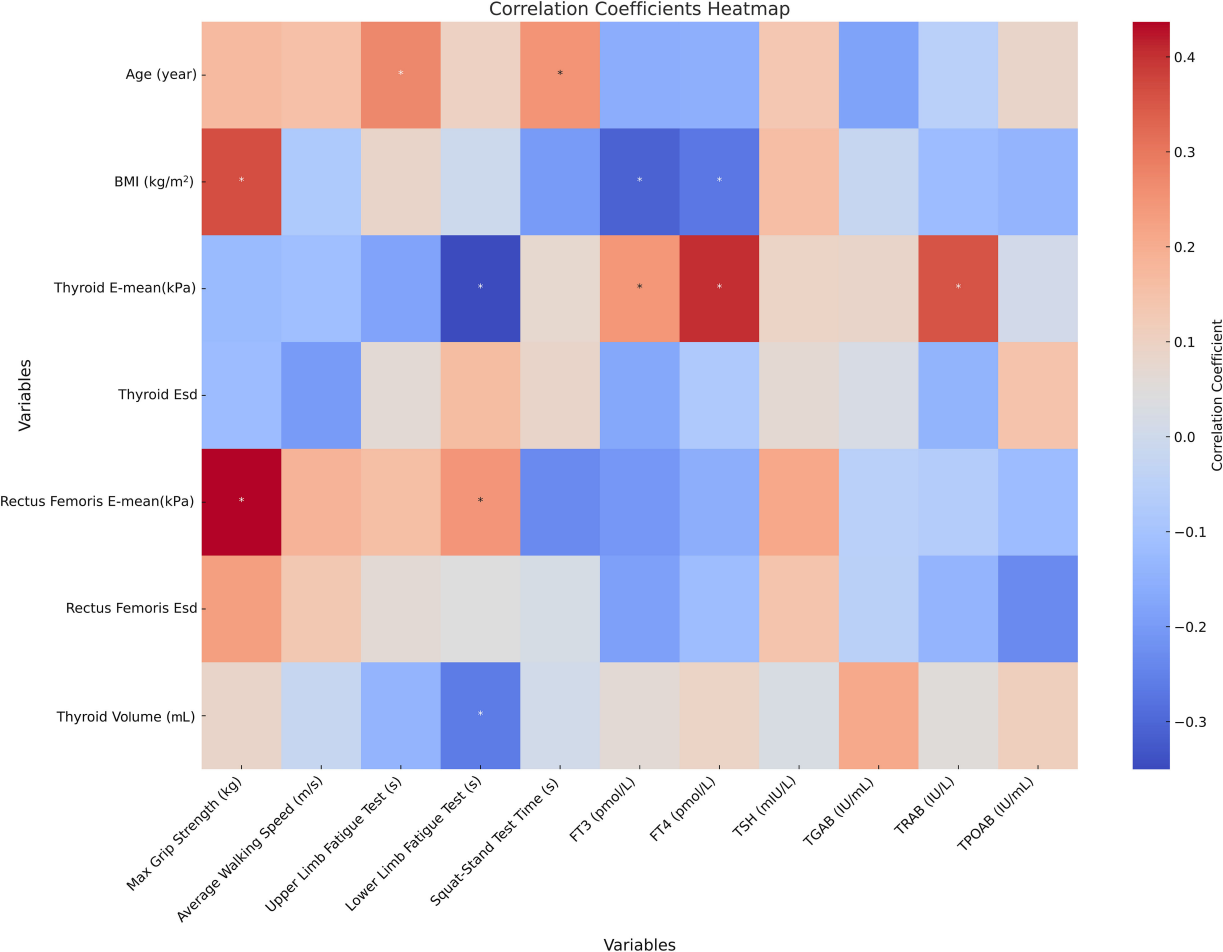
Correlation analysis of thyroid indicators, skeletal muscle function, strength tests, and elasticity indicators in patients with CTM ^*^ represents *p* < 0.05; Correlation analysis reflects only the statistical associations between data or indicators. BMI, body mass index; CTM, chronic thyrotoxic myopathy; E-mean, mean of Young’s modulus; Esd, elastic data dispersion; FT3, free triiodothyronine; FT4, free thyroxine; TSH, thyroid-stimulating hormone; TGAB, thyroglobulin antibody; TRAB, thyrotropin receptor antibody; TPOAB, thyroid peroxidase antibodies.

Correlation analysis revealed that age was significantly positively correlated with the upper limb fatigue test (r = 0.274, *p* < 0.05) and squat–stand test (r = 0.251, *p* < 0.05). Body mass index (BMI) was positively correlated with grip strength (r = 0.364, *p* < 0.01) but negatively correlated with FT3 (r = −0.309, *p* < 0.05) and FT4 (r = −0.270, *p* < 0.05). The thyroid E-mean was negatively correlated with the lower limb fatigue test (r = −0.350, *p* < 0.01) but positively correlated with FT3 (r = 0.245, *p* < 0.05), FT4 (r = 0.404, *p* < 0.01), and TRAB (r = 0.356, *p* < 0.01). The rectus femoris E-mean was positively correlated with grip strength (r = 0.437, *p* < 0.01) and lower limb fatigue test (r = 0.247, *p* < 0.05). Additionally, thyroid volume was negatively correlated with the lower limb fatigue test (r = −0.263, *p* < 0.05; **Figure 4**).

### 3.6 Changes in body composition and muscle quality after treatment

**Figure 5:**
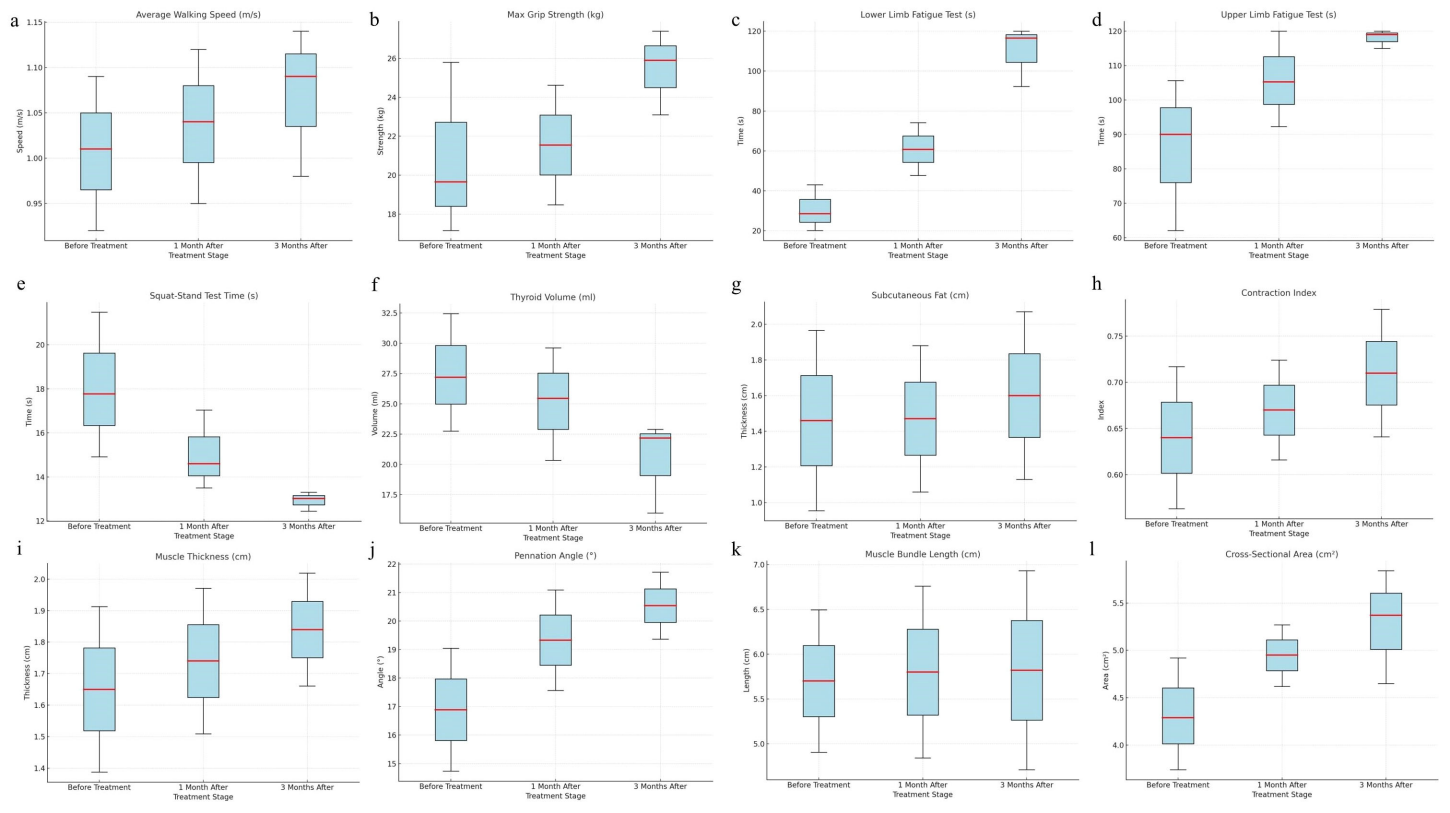
Changes in skeletal muscle strength, function, and muscle quality indicators in patients with CTM (before treatment, 1 month after treatment, and 3 months after treatment) **(a)** Average walking speed, **(b)** maximum grip strength, **(c)** lower limb fatigue test, **(d)** upper limb fatigue test, **(e)** squat–stand test time, **(f)** thyroid volume, **(g)** subcutaneous fat, **(h)** contraction index, **(i)** muscle thickness, **(j)** pennation angle, **(k)** muscle bundle length, **(l)** cross-sectional area.

**Figure 6:**
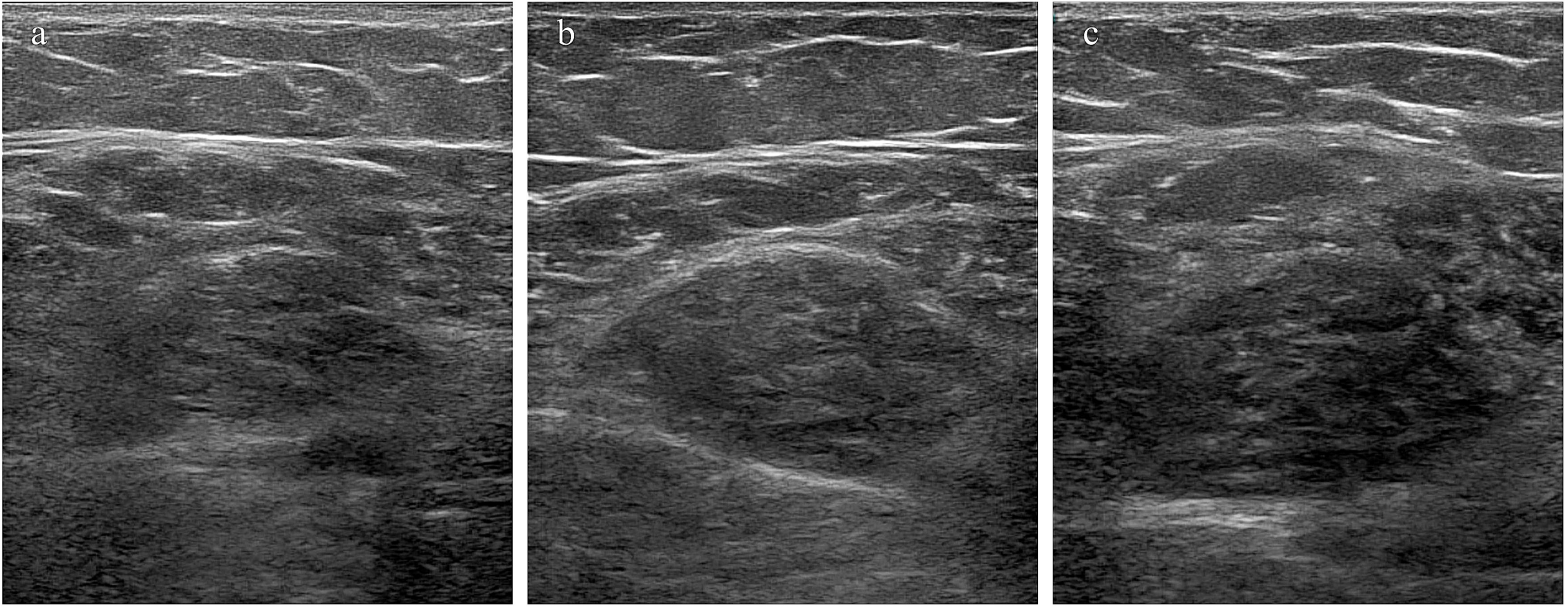
Changes in muscle cross-sectional area following treatment. **(a–c)** Muscle cross-sectional area in a 40-year-old female patient before treatment, 1 month after treatment, and 3 months after treatment.

The follow-up results demonstrated that patients with CTM exhibited significant improvements in upper and lower limb fatigue tests and functional indicators (squat–stand test, average walking speed, and maximum grip strength) following treatment (*p* < 0.001), indicating improved endurance and movement efficiency. Thyroid function indicators (FT3 and FT4) significantly decreased, whereas thyroid stimulating hormone (TSH) gradually recovered (*p* < 0.001), suggesting effective thyroid function regulation, and reduced thyroid volume may reflect pathological improvement (**Supplementary Figure S1**). Post-treatment increases in BMI and waist, hip, and thigh circumferences were observed, indicating weight gain because of slowed metabolism following hyperthyroidism control; however, the waist-to-hip ratio showed no significant change (*p* > 0.05). The heart rate significantly decreased, with all patients achieving a heart rate of <100 beats/min (bpm) after 3 months of treatment, yielding a 100% remission rate (*p* < 0.01). The basal metabolic rate significantly decreased, falling below 15 KJ/(m^2^·h) after 3 months, primarily associated with decreased thyroid hormone levels. No significant changes in systolic or diastolic blood pressure were observed (*p* > 0.05) (**Supplementary Table S1**). Moreover, the post-treatment contraction index significantly increased (*p* < 0.05), muscle thickness and cross-sectional area improved (*p* = 0.050, *p* = 0.018), and pennation angle significantly increased (*p* < 0.001) (**Supplementary Table S2**). In summary, pharmacological treatment in patients with CTM significantly improved skeletal muscle function, thyroid function, and muscle quality, while also exerting beneficial effects on metabolic and cardiovascular indicators (**Figures 5**, **6**).

## 4 Discussion

In this study, the biomechanical properties of the rectus femoris and thyroid tissues in patients with CTM were systematically evaluated using a multimodal ultrasonographic approach. We developed an early predictive model for CTM by incorporating SWE parameters of the thyroid and rectus femoris muscles. The results demonstrated that patients with CTM exhibited significantly lower E-mean values in the rectus femoris than both non-CTM and healthy controls, suggesting that fat accumulation in the muscles may represent a key pathological hallmark of the disease. Concurrently, a marked elevation in the E-mean of thyroid tissue was observed, indicating pronounced inflammatory infiltration and fibrotic remodeling. CTM, a muscle disorder, is closely linked to hyperthyroidism and is characterized by progressive muscle weakness, atrophy, and emaciation. The disease onset is frequently insidious, with gradual progression (12). Current research indicates that the pathogenesis of CTM involves the direct stimulatory effects of thyroid hormones on skeletal muscle and the dysregulation of the regulatory mechanisms of muscle tissue (13, 14). The interaction between thyroid hormones, insulin-like growth factor 1, and autophagy processes is believed to play a crucial role in muscle development and functional maintenance (15). Genetic susceptibility, autoimmune responses, and environmental factors play significant roles in CTM onset and progression (16). Although the pathophysiological mechanisms underlying CTM are not fully understood, its substantial effects on patients’ quality of life makes it a major clinical concern. Accordingly, the use of ultrasonographic technology for assessing muscle mass has recently emerged as a focal point of research (17–19). UE has key advantages in evaluating muscle disease severity, monitoring treatment effects, and supporting long-term follow-up by quantifying dynamic changes in muscle stiffness before and after treatment (20). In this study, we optimized the measurement protocol, including multiple measurements, elastography mode selection, probe choice, data post-processing, and multimodal imaging integration. This strategy effectively mitigated the effects of tissue anisotropy on elastography results, significantly enhancing measurement accuracy and reproducibility.

This study used SWE to assess the rectus femoris and thyroid tissues in patients with CTM. For muscle assessment, we selected the superficial rectus femoris as the target muscle and noted that patients with CTM exhibited significantly lower rectus femoris E-mean values than the non-CTM and healthy control groups. This finding aligns with the propagation characteristics of shear waves in biological tissues: shear waves propagate faster in stiffer tissues and slower in softer tissues (21). The pathological mechanism underlying the decreased rectus femoris stiffness in patients with CTM may be similar to that of sarcopenia, and the potential mechanism could be strongly associated with ectopic fat deposition within muscle tissues. Ectopic fat deposition may disrupt the microstructure of muscle fibers, reduce the mechanical properties of muscle tissue, and impair muscle contractile function, ultimately causing a significant decrease in muscle stiffness (22). This pathological mechanism has been broadly investigated and validated in sarcopenia; however, its specific role in CTM and its effect on disease progression warrant further exploration. Notably, the rectus femoris E-mean was positively correlated with the grip strength and lower limb fatigue test, suggesting a correlation between muscle stiffness and skeletal muscle function and strength. Furthermore, this study is the first to explore the application of the Esd in CTM assessment. As a quantitative indicator of the heterogeneity of elasticity modulus, Esd can reflect the heterogeneity of tissue components. Our results revealed significantly reduced Esd values in patients with CTM, suggesting decreased muscle tissue component uniformity. This finding offers new insights into the pathophysiological mechanisms of CTM. Currently, studies on Esd in muscle diseases are limited, and the findings of this study provide crucial reference points for the clinical application of this parameter. Furthermore, this study used SWE to quantitatively analyze thyroid stiffness in patients with CTM. Normal thyroid tissues comprise homogeneous follicular structures and a rich network of capillaries, providing it a relatively soft texture. However, under pathological conditions, follicular disruption, inflammatory cell infiltration, and fibrous tissue proliferation can result in significantly increased tissue stiffness (23). In this study, patients with CTM demonstrated a significantly higher average Young’s modulus (E-mean) of the thyroid than the non-CTM and healthy control groups, consistent with previous findings of increased thyroid stiffness in patients with hyperthyroidism (24), suggesting that patients with CTM can exhibit more pronounced thyroid inflammatory infiltration and fibrosis. The thyroid E-mean was positively correlated with FT3, FT4, and TRAB, suggesting a correlation between thyroid hormone, TRAB, and thyroid hardness. Furthermore, the CTM group had a significantly larger thyroid volume than the non-CTM group, which may be attributed to a higher degree of thyroid follicular cell and vascular proliferation. Notably, the ROC curve analysis revealed that the combined Young’s modulus parameters of the rectus femoris and thyroid provided significantly better predictive performance for CTM than individual markers, with an AUC of 92.5%. This finding indicates that multimodal ultrasonography demonstrates high sensitivity and specificity in the early diagnosis of CTM. Multivariate logistic regression further confirmed that the Young’s modulus values of both the rectus femoris (E-mean) and thyroid (E-mean) are independent factors associated with CTM, providing objective evidence for early disease prediction. These findings align with those of previous studies, supporting the use of SWE for the real-time quantitative assessment of tissue stiffness changes and providing reliable guidance for the diagnosis and staging of diffuse thyroid diseases (11, 25). Recently, the clinical utility of UE in thyroid research has attracted considerable attention, particularly in the context of early diagnosis and therapeutic decision-making. A recent study has demonstrated that the combination of elastography with fine-needle aspiration enhances diagnostic confidence for thyroid nodules (26). In addition, elastography is effective for conventional thyroid nodule evaluation as well as for diagnosing lesions with indeterminate cytology or subcentimeter size (27). Collectively, these studies indicate that UE, by enabling the quantitative detection of subtle changes in tissue stiffness, holds promise as an important adjunct tool for early risk stratification, therapeutic planning, and longitudinal monitoring in thyroid disease. Furthermore, these findings support the rationale of applying SWE to assess thyroid stiffness in patients with CTM, thereby expanding the diagnostic relevance of elastography within a multimodal ultrasound framework.

We also investigated changes in clinical parameters following antithyroid drug treatment in patients with CTM. The results revealed that patients experienced significant increases in BMI and waist, hip, and thigh circumferences following treatment, suggesting fat accumulation and weight gain. This finding is consistent with that of a previous study (28), and the underlying mechanism may be associated with reduced metabolic rate, insulin resistance, and thyroid hormone level normalization following the control of hyperthyroidism (29, 30). Thyroid function tests revealed that, after the 3-month treatment, most patients demonstrated restored normal FT3 and FT4 levels. However, five patients still had TSH levels below the normal range, and a few exhibited hypothyroidism-related symptoms. In this study, 91% of patients had their heart rate controlled to <100 bpm within 1 month following treatment, and all patients had their heart rate restored to the normal range within 3 months. Thyroid hormone levels gradually normalized as the treatment medication took effect, decreasing cardiac stimulation and significantly improving the heart rate. Therefore, in patients with CTM, antithyroid drug treatment can effectively improve thyroid function and cardiovascular symptoms. However, caution is warranted regarding post-treatment weight gain and the potential risk of hypothyroidism. These findings offer significant insights into the clinical management of these patients. Moreover, in this study, patients with CTM exhibited significantly increased subcutaneous fat thickness, muscle thickness, pennation angle, fascicle length, muscle cross-sectional area, and contraction index of the vastus rectus muscle following treatment, indicating a gradual improvement in muscle atrophy. This finding aligns with those of previous studies on sarcopenia management (31). The continuous increase in muscle thickness and cross-sectional area following treatment indicates that muscle atrophy has progressively improved. Xifra et al. (32) reported a significant recovery of muscle mass following successful treatment of hyperthyroidism. However, some studies have highlighted that fat synthesis predominates in the early stages of treatment (the first 2 months), whereas muscle mass increase becomes more significant in the later stages (the third month) (33). Overall, our findings demonstrate that antithyroid drug treatment can significantly improve the symptoms of muscle atrophy in patients with CTM. Based on the observed structural improvements, we further assessed grip strength and walking speed, which are recognized indicators of skeletal muscle strength, to evaluate muscle function in CTM patients. The results showed significantly increased post-treatment grip strength compared with that before treatment, indicating that antithyroid therapy can effectively improve upper limb muscle strength. This finding aligns with those of Zhou et al. (34). Furthermore, before treatment, patients with CTM showed significantly lower walking speed than the non-CTM and healthy groups; however, it did not meet the diagnostic criteria for sarcopenia. This discrepancy may be due to factors, including emotional fluctuations, that could interfere with the assessment. Post-treatment, improvements in upper and lower limb fatigue tests and the squat–stand test reflected lower limb muscle function and coordination recovery. Improvements in grip strength and walking speed further confirmed enhanced muscle strength and function in these patients. Therefore, antithyroid therapy can significantly alleviate muscle weakness symptoms in patients with CTM. However, the existing diagnostic criteria for sarcopenia may not fully apply to patients with CTM. Future studies should develop a more precise muscle function evaluation system specifically for CTM to optimize clinical diagnostic and treatment strategies.

Several limitations should be acknowledged. First, we could not categorize participants based on their daily physical activity levels or occupation. Second, the follow-up of patients with CTM was limited because of the relatively short study duration, and some findings were not included in this analysis. Moreover, we could not comprehensively include information on chronic comorbidities, inflammation-related laboratory parameters, and history of thyroid medication use among patients because of limitations in clinical data collection and the inclusion and exclusion criteria. This may have affected a more in-depth exploration of the pathogenesis of CTM and its ultrasonographic characteristics. Future studies should incorporate and validate these factors to further deepen and broaden the scope of research. Third, the present study only assessed the rectus femoris muscle based on its anatomical advantages and superior measurement repeatability in preliminary trials. However, the exclusion of other quadriceps components—such as the vastus lateralis or vastus medialis—may have limited the generalizability of our findings to broader muscle groups. Future studies should consider employing more advanced imaging techniques or improved standardization protocols to evaluate additional muscle sites, thereby validating and extending the applicability of the results.

Nevertheless, this study still has significant clinical relevance. We conducted multimodal quantitative analyses of the rectus femoris and thyroid tissues in patients with CTM using high-frequency ultrasonography combined with SWE, revealing that muscle and thyroid tissue stiffness were strongly associated with the risk of CTM onset. The results demonstrated that patients with CTM exhibited a significantly lower rectus femoris E-mean than the non-CTM and healthy control groups, suggesting that fat accumulation in the muscles could be a key pathological feature of CTM. Therefore, this study systematically evaluated the biomechanical characteristics of muscle and thyroid tissues in patients with CTM using multimodal ultrasonography, along with indicators such as skeletal muscle function, strength metrics, and thyroid hormones. An early predictive model was also developed, further confirming the clinical utility of this approach for treatment monitoring. To optimize diagnostic and treatment strategies, future studies should expand the sample size and explore additional CTM-related biomechanical parameters.

## 5 Conclusion

This study revealed that reduced rectus femoris muscle stiffness and increased thyroid gland stiffness are characteristic changes that serve as key diagnostic indicators for CTM. The significant correlation between the E-mean value of the rectus femoris and skeletal muscle function parameters along with the high accuracy of combined UE parameters (AUC = 92.5%) further highlights the clinical value of multimodal ultrasonography technology in the early screening and disease monitoring of CTM.

## Data Availability

The original contributions presented in the study are included in the article/**Supplementary Material**. Further inquiries can be directed to the corresponding author.
