# Supplementary material for: Multimodal ultrasonography in the diagnosis and treatment of chronic thyrotoxic myopathy: a prospective study
Source: Front Endocrinol (Lausanne). 2025 Sep 11;16:1590294. doi: 10.3389/fendo.2025.1590294 (PMC12460117; doi:10.3389/fendo.2025.1590294)
Supplement: Supplementary file 1 [file DataSheet1.docx]

**Table S1: Comparison of Baseline Characteristics in CTM Patients Before and After Treatment**

| **Characteristic** | **Before Treatment** | **1 Month After Treatment** | **3 Months After Treatment** | ***p*** |
| --- | --- | --- | --- | --- |
| BMI  (kg/m^2^) | 20.53 ± 2.62 | 20.55 ± 2.639 | 20.71 ± 1.792 | 0.738 |
| Systolic Blood Pressure (mmHg) | 125.30 ± 15.776 | 120.10 ± 11.998 | 119.23 ± 13.192 | 0.283 |
| Diastolic Blood Pressure (mmHg) | 74.00 (69.00, 79.00) | 71.50 (69.75, 76.25) | 73.00 (71.00, 81.00) | 0.470 |
| Pulse Pressure(mmHg) | 54.00 (47.25, 61.50) | 46.00 (41.00, 55.00) | 40.00 (40.00, 48.00) | 0.021 |
| Heart Rate (beats/min) | 107.00 (100.25, 120.75) | 79.00 (68.50, 91.00) | 73.00 (70.00, 74.00) | <0.001 |
| Basal Metabolic Rate [KJ/(m^2^·h)] | 52.13 ± 19.470 | 17.75 ± 16.257 | 5.00 ± 9.687 | <0.001 |
| Waist Circumference (cm) | 73.56 ± 6.295 | 74.70 ± 7.147 | 76.42 ± 6.689 | 0.182 |
| Hip Circumference (cm) | 87.25 (83.53, 90.50) | 88.50 (83.75, 92.00) | 86.00 (85.00, 91.00) | 0.875 |
| Waist-to-Hip Ratio | 0.85 ± 0.048 | 0.86 ± 0.048 | 0.87 ± 0.046 | 0.053 |
| Thigh Circumference (cm) | 45.50 (43.50, 50.00) | 47.10 (45.00, 50.03) | 47.00 (46.50, 51.00) | 0.173 |

Note：BMI: Body Mass Index.

**Table S2: Comparison of Muscle Mass in CTM Patients Before and After Treatment**

| **Characteristic** | **Before Treatment** | **1 Month After Treatment** | **3 Months After Treatment** | ***p*** |
| --- | --- | --- | --- | --- |
| Contraction Index | 0.64 ± 0.077 | 0.67 ± 0.054 | 0.71 ± 0.069 | 0.011 |
| Subcutaneous Fat (cm) | 1.46 ± 0.506 | 1.47 ± 0.410 | 1.60 ± 0.47 | 0.647 |
| Subcutaneous Fat (cm) | 1.65 ± 0.263 | 1.74 ± 0.231 | 1.84 ± 0.179 | 0.050 |
| Pennation Angle (°) | 16.89 ± 2.156 | 19.33 ± 1.762 | 20.54 ± 1.174 | <0.001 |
| Muscle Bundle Length (cm) | 5.70 ± 0.795 | 5.80 ± 0.959 | 5.82 ± 1.110 | 0.897 |
| Cross-Sectional Area (cm^2^) | 4.40±0.99 | 5.03±0.99 | 5.39±1.30 | 0.018 |

**
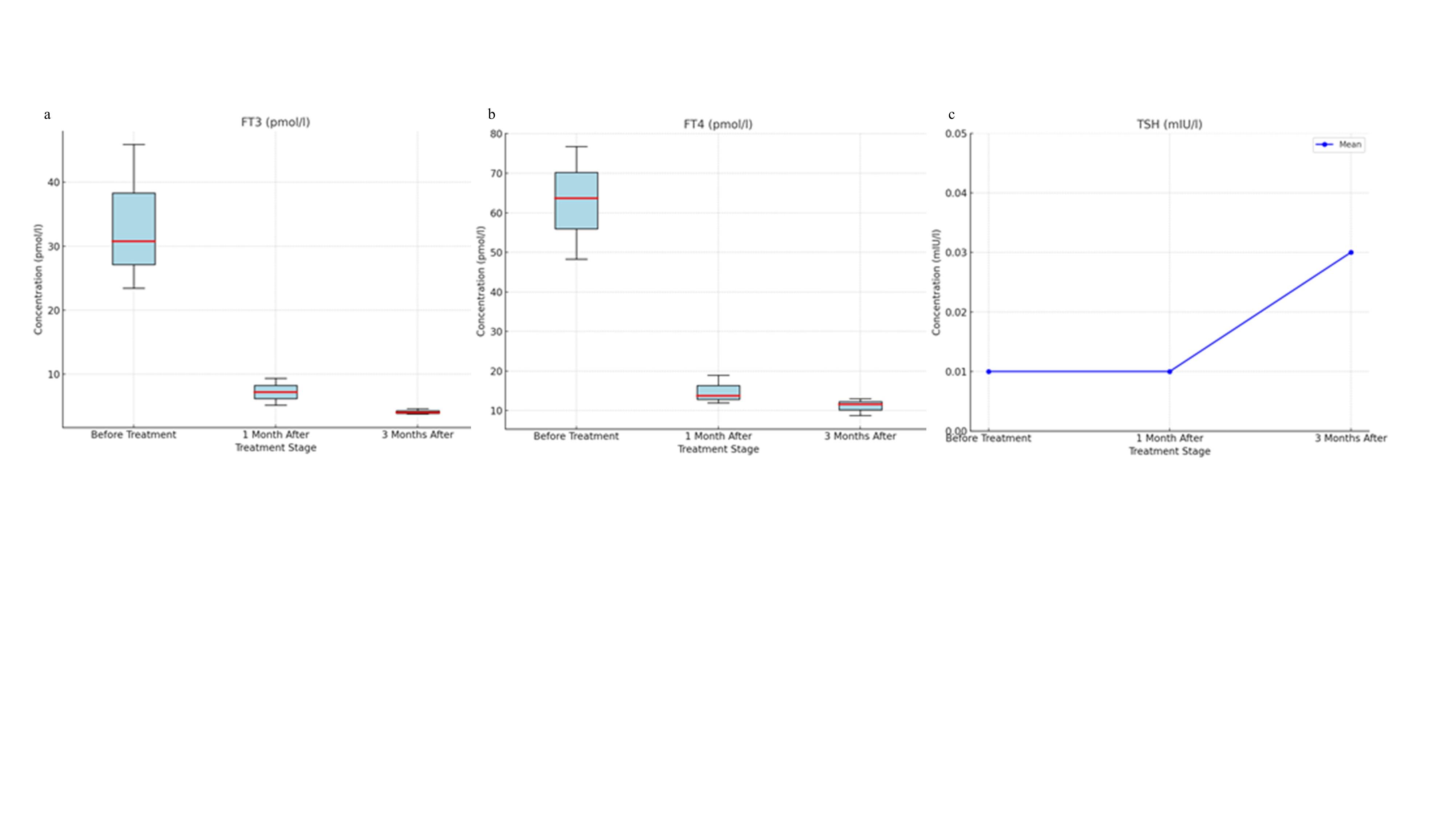
**

**Figure S1: Comparison of Thyroid Function in CTM Patients Before and After Treatment.** Note：FT3: Free Triiodothyronine; FT4: Free Thyroxine; TSH: Thyroid Stimulating Hormone.
